# Graphene Substrates Promote the Differentiation of Inner Ear Lgr5+ Progenitor Cells Into Hair Cells

**DOI:** 10.3389/fbioe.2022.927248

**Published:** 2022-06-22

**Authors:** Xiaoqiong Ding, Yangnan Hu, Hong Cheng, Xiaoli Zhang, Ling Lu, Song Gao, Cheng Cheng, Lifen Wang, Xiaoyun Qian, Chen Zhang, Renjie Chai, Xia Gao, Zhichun Huang

**Affiliations:** ^1^ Department of Otorhinolaryngology-Head and Neck Surgery, Nanjing Drum Tower Hospital, Drum Tower Clinical College of Nanjing Medical University, Nanjing, China; ^2^ Department of Otorhinolaryngology-Head and Neck Surgery, Zhongda Hospital, Southeast University, Nanjing, China; ^3^ State Key Laboratory of Bioelectronics, Jiangsu Province High-Tech Key Laboratory for Bio-Medical Research, School of Life Sciences and Technology, Advanced Institute for Life and Health, Southeast University, Nanjing, China; ^4^ Department of Otorhinolaryngology-Head and Neck Surgery, Affiliated Drum Tower Hospital of Nanjing University Medical School, Nanjing, China; ^5^ Beijing Key Laboratory of Neural Regeneration and Repair, Capital Medical University, Beijing, China; ^6^ Department of Otolaryngology Head and Neck Surgery, Sichuan Provincial People’s Hospital, University of Electronic Science and Technology of China, Chengdu, China; ^7^ Co-Innovation Center of Neuroregeneration, Nantong University, Nantong, China; ^8^ Institute for Stem Cell and Regeneration, Chinese Academy of Science, Beijing, China

**Keywords:** graphene, sensorineural hearing loss, hair cell regeneration, proliferation, differentiation

## Abstract

The ideal treatment for sensory hearing loss is to regenerate inner ear hair cells (HCs) through stem cell therapy, thereby restoring the function and structure of the cochlea. Previous studies have found that Lgr5+ supporting cells (SCs) in the inner ear can regenerate HCs, thus being considered inner ear progenitor cells. In addition to traditional biochemical factors, physical factors such as electrical conductivity also play a crucial role in the regulation of stem cell proliferation and differentiation. In this study, the graphene substrates were used to culture Lgr5+ progenitor cells and investigated their regulatory effects on cells. It was demonstrated that the graphene substrates displayed great cytocompatibility for Lgr5+ progenitors and promoted their sphere-forming ability. Moreover, more Myosin7a+ cells were found on the graphene substrates compared with tissue culture polystyrene (TCPS). These results suggest that graphene is an efficient interface that can promote the differentiation of Lgr5+ progenitors into HCs, which is great significance for its future application in combination with Lgr5+ cells to regenerate HCs in the inner ear.

## Introduction

Inner ear sensory hair cells (HCs) mainly function for transduction of mechanical stimuli into electrical signals and are mechanoreceptors for sound recognition (Li et al., 2018; Liu et al., 2019; Zhang et al., 2019). Aging, ototoxic drugs, trauma, inflammation, and other factors can all contribute to hair cell damage, resulting in sensorineural hearing loss (Liu et al., 2016; Zhou et al., 2020). Sensorineural hearing loss is a common sensory disease caused by damage or loss of HCs, affecting millions of people worldwide. Supporting cells (SCs) in the auditory and vestibular systems of birds and fish have been reported to have the capability to regenerate HCs in response to the damage to HCs (Corwin and Cotanche, 1988; Balak et al., 1990; Warchol, 2011). However, studies have shown that HC damage in adult mammals is irreparable, which lead to permanent hearing loss (Rubel et al., 2013). Recently, several studies reported that Lgr5+ SCs in the inner ear can regenerate HCs and considered inner ear progenitor cells, which bringing new possibilities for the regeneration of HCs in adult mammals (Bermingham-McDonogh and Reh, 2011; Chai et al., 2011; Chai et al., 2012; Shi et al., 2013; Bramhall et al., 2014; Waqas et al., 2016).

The most ideal treatment for sensory hearing loss is to regenerate inner ear HCs to restore the structure and function of the cochlea (Wang et al., 2015). The behaviors of cochlear of progenitor cells including proliferation and differentiation are regulated by numerous biochemical and physical factors. Investigating the regulatory strategies that promote the differentiation of cochlear progenitor cells into mature HCs is critical for HC regeneration and hearing reconstruction. Several studies have shown that some conductive materials such as graphene and MXenes can regulate the behavior of stem cells, including proliferation and differentiation (Guo et al., 2016; Farokhi et al., 2021; Yao et al., 2021; Guo et al., 2022). Lgr5+ progenitors have characteristics similar to adult stem cells. The detailed regulatory effects of these conductive materials on Lgr5+ progenitors’ behavior and their potential in the treatment of sensorineural hearing loss have not been investigated.

Herein, in this work, we fabricated graphene substrates and studied their regulatory effects on the proliferation and differentiation. Graphene has been reported to significantly promote neuronal differentiation of neural stem cells (NSCs). Several studies have reported their potential for applications in the biomedical field, including drug delivery (Song et al., 2020; Sattari et al., 2021), photothermal therapy (de Melo-Diogo et al., 2019; Palmieri et al., 2020; Wang et al., 2020), and nerve regeneration (Aydin et al., 2018; Grijalvo and Díaz, 2021). Therefore, the current study focus on the effects of graphene substrate on the survival, proliferation and differentiation into HCs in Lgr5+ progenitors, which is of great significance for the combined therapy of physical stimulation and stem cell transplantation for the treatment of sensorineural hearing loss.

## Materials and Methods

### Animals

Lgr5-EGFP-IRES-creERT2 mice (stock no. 008875) were purchased from The Jackson Laboratory. All animal experiments were performed in accordance with protocols that were approved by the Animal Care and Use Committee of Southeast University (Approved No. 20200402025) and the National Institute of Health’s Guide for the Care and Use of Laboratory Animals. All efforts were made to minimize the number of animals used and to prevent their suffering.

### Genotyping PCR

For genotyping of transgenic mice, the tail tips were first collected, and genomic DNA was obtained by adding 180 uL 50 mM NaOH and incubating at 98°C for 1 h, followed by adding 20 uL 1 M Tris-HCl (pH 7.0). The tube was then vortexed vigorously for 1 min for complete tissue dispersion. The primers used for genotyping are as follows; wild-type (R) ATA CCC CAT CCC TTT TGA GC; Lgr5: (F) CTG CTC TCT GCT CCC AGT CT; mutant (R) GAA CTT CAG GGT CAG CTT GC.

### Preparation and Characterization of Graphene Substrates

The graphene substrates were purchased from Nanjing MKNANO Tech. Co., Ltd. Specifically, the graphene was prepared by chemical vapor deposition (CVD) and then transferred to the surface of coverslips to obtain a graphene substrate. For cell culture, the graphene substrates were immersed in 75% alcohol for 1 h, then washed three times with sterilized water and irradiated under ultraviolet light overnight.

The surface of graphene substrates was observed by Scanning Electron Microscope (SEM) (Zeiss, Ultra Plus). The graphene substrates were then characterized by a Raman microscopy (Renishaw inVia) and an X-ray diffractometer (XRD) (PANalytical Empyrean).

### Isolation of Lgr5+ Cels by Flow Cytometry

The cochleae of Lgr5-EGFP-creERT2 mice (postnatal day 1–3, P1-3) were dissected out and collected in a tube. Subsequently, the collected cochleae were digested by trypsin (0.125%, Invitrogen) at 37°C for 8 min, followed by adding equal volume of soybean trypsin inhibitor (Worthington Biochem) to terminate the digestion. Cochleae tissue were dissociated into single cell suspensions by blunt tips (Eppendorf) and then filtered with a 40 μm cell strainer (BD Biosciences). Finally, the obtained single cells were sorted by a BD FACS Aria III (BD Biosciences) through GFP channel. Finally, the sorted cells were cultured on graphene substrates and tissue culture polystyrene (TCPS), respectively.

### Sphere-Forming Assay and Differentiation Assay

The cells sorted by FAC were cultured in DMEM/F12 medium supplemented with N2 (1%, Invitrogen), B27 (2% dilution, Invitrogen), EGF (20 ng/ml, Sigma), bFGF (10 ng/ml, Sigma), and IGF-1 (50 ng/ml, Sigma), heparin sulfate (50 ng/ml, Sigma) (full medium), and ampicillin (0.1%, Beyotime). For sphere-forming assay, the sorted cells were seeded on TCPS or graphene substrates with 200 cells per well for 5 days. Subsequently, we measured the number and diameter of the formed spheres to evaluate their proliferation capacity. For differentiation assay, the sorted single cells and formed spheres were differentiated separately. The cells or spheres were cultured in a 4-well dish for 10 days, and immunofluorescence staining was then applied to analyze the differentiation of Lgr5+ cells. As described in previous reports, cell aggregation with more than 5 cells were considered a sphere or colony.

### Immunostaining and Image Acquisition

After culture, the cells grown on different substrates were fixed with 4% paraformaldehyde for 1 h at room temperature and then washed with PBS containing 0.1% Triton X-100 (PBST). The cells were then blocked with PBS containing 1% BSA for 1 h at room temperature. Afterwards, the primary antibody anti-Myosin7a (Proteus Bioscience) was diluted and incubated with these cells at 4°C overnight. After that, the cells were washed with PBST and then incubated with secondary antibody along with 2-(4-Amidinophenyl)-6-indolecarbamidine dihydrochloride (DAPI) for 1 h at room temperature. Finally, the cells cultured on different substrates were washed and then covered with coverslips in fluorescence mounting medium (DAKO). The cells were observed and captured with a laser scanning confocal microscope (Zeiss, LSM 700).

### Statistical Analysis

All data were analyzed with GraphPad Prism6 software and presented as mean ± SD. Two-tailed, unpaired Student’s t-tests were used to determine statistical significance when comparing two groups, and a value of *p* < 0.05 was considered statistically significant. At least three individual experiments were conducted for all experiments.

## Results

### Characterization of Graphene

**FIGURE 1:**
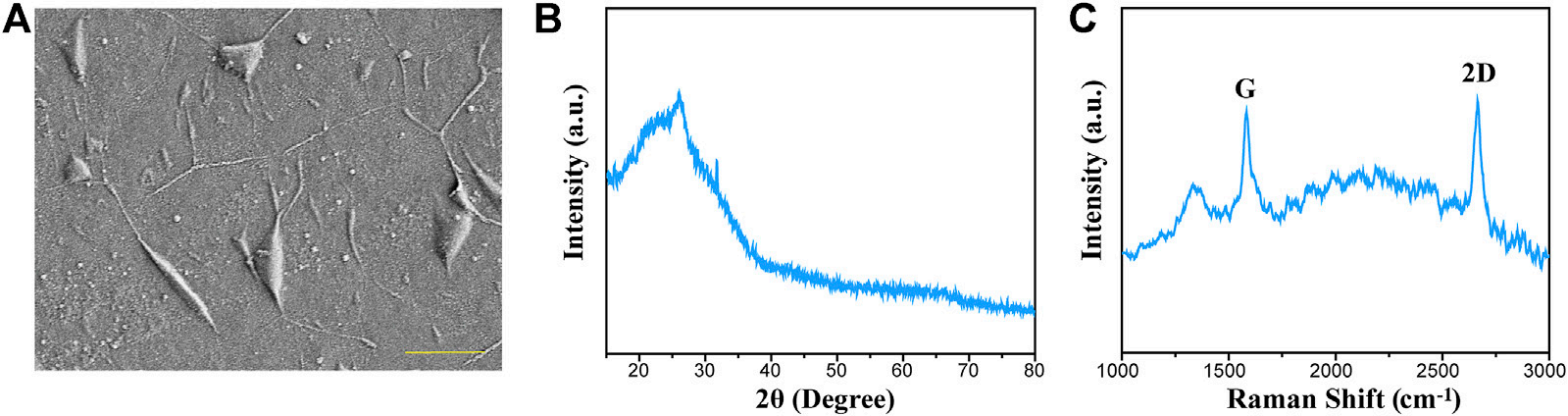
Characterization of graphene substrates prepared by the CVD method. **(A)** Representative SEM image of the graphene substrates. **(B)** XRD spectra for graphene substrates. **(C)** Raman spectra for graphene substrates.

Graphene substrates used in our work were fabricated by a CVD method and then transferred to the surface of coverslips. The morphology of the graphene substrates was observed from the SEM image (Figure 1A). Figure 1B displays the XRD pattern of graphene substrates. It was suggested that the graphene has a distinct peak at 27.5°, which can be indexed to (002) diffraction plane. The Raman spectra displayed the characteristic peaks of 2D and G bands, and the intensity ratio indicated that the graphene substrates composed of a few layers of graphene sheets (Figure 1C).

### Graphene Substrates Have No Effects on the Viability of Lgr5+ Cells

**FIGURE 2:**
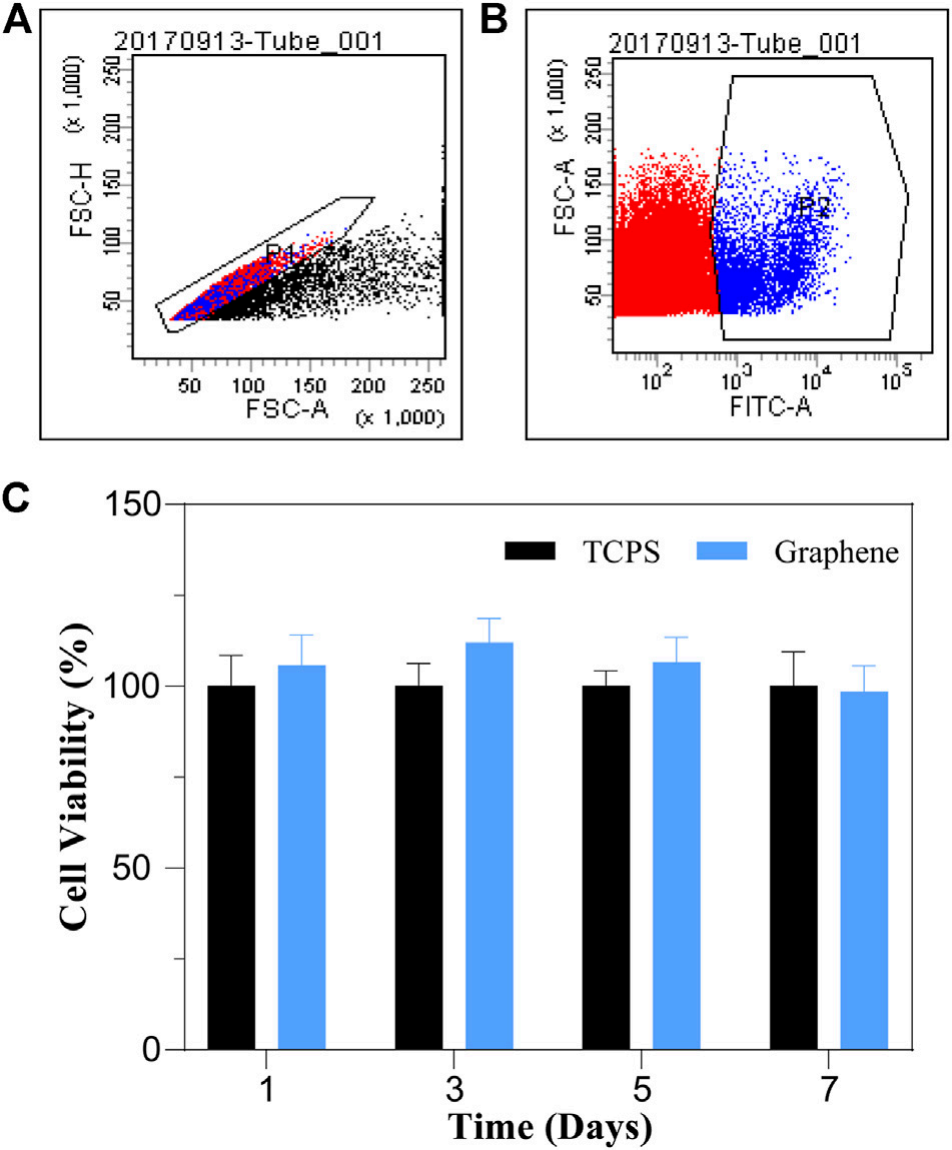
FAC sorting plots and the effects of graphene substrates on the cell viability of Lgr5+ progenitors. **(A,B)** FAC sorting plots. **(C)** Cell viability results from CCK-8 assay.

To evaluate the cytocompatibility of graphene substrates, we isolated Lgr5+ cells from Lgr5-EGFP- CreERT2 mice by FAC sorting and cultured the sorted cells on graphene substrates and TCPS for different times. The flow cytometry plots displayed that about 5.8% of the whole cochlear cell population were Lgr5+ progenitors (Figures 2A,B). After culture, a CCK-8 assay was conducted to analyze the influence of graphene substrates on the viability of Lgr5+ cells (Figure 2C). It was suggested that cells cultured on graphene substrates for different times had similar viability to cells cultured on TCPS, indicating our prepared graphene substrates are nontoxic and cytocompatible. Therefore, we further investigated the effects of graphene substrates on the proliferation and differentiation of Lgr5+ progenitors.

### Lgr5+ Progenitors Cultured on Graphene Substrates Have Higher Sphere-Forming Ability Than Those Cultured on TCPS

**FIGURE 3:**
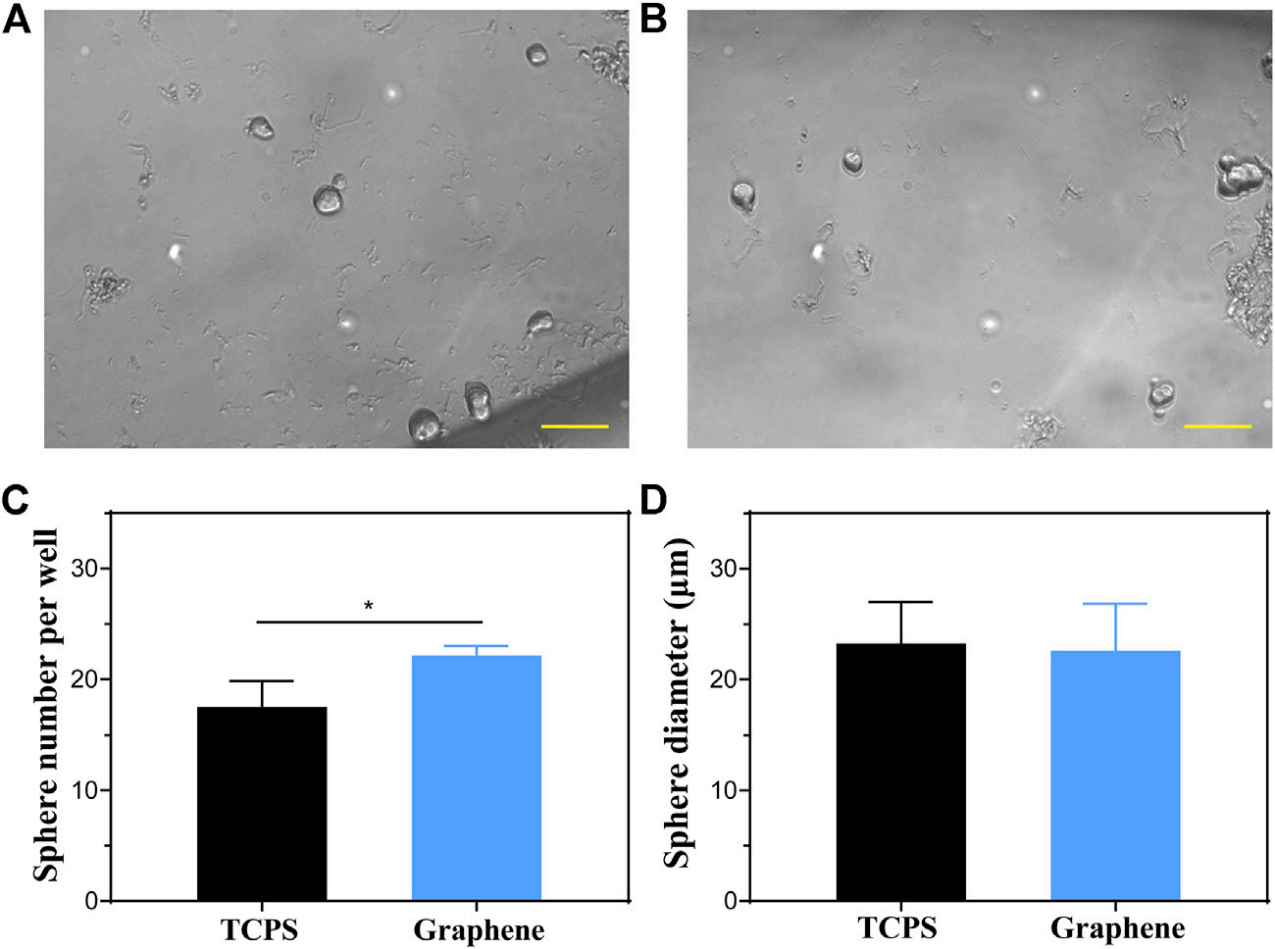
Sphere-forming assay of Lgr5+ progenitors. **(A,B)** The spheres formed by Lgr5+ progenitors cultured on TCPS **(A)** and graphene substrates **(B)**. **(C,D)** The sphere number **(C)** and diameter **(D)** of Lgr5+ spheres cultured on different substrates. The scale bars are 50 μm in **(A,B)**.

The capacity of self-renew is an important characteristic of stem cells and progenitor cells. Therefore, we cultured the sorted cells on graphene substrates and TCPS for 5 days to form spheres, respectively, to determine their proliferation capacity. Figures 3A,B showed images of spheres formed from Lgr5+ progenitors cultured on different substrates. It was suggested that the Lgr5+ progenitors cultured on graphene substrates formed more spheres than those cultured on TCPS (Figure 3C). However, there was no significant difference in the diameter of the formed spheres (Figure 3D). These results demonstrated that our prepared graphene substrates could promote the sphere-forming ability of Lgr5+ progenitors.

### Lgr5+ Progenitors Cultured on Graphene Substrates Generate More HCs Compared to Those Cultured on TCPS

**FIGURE 4:**
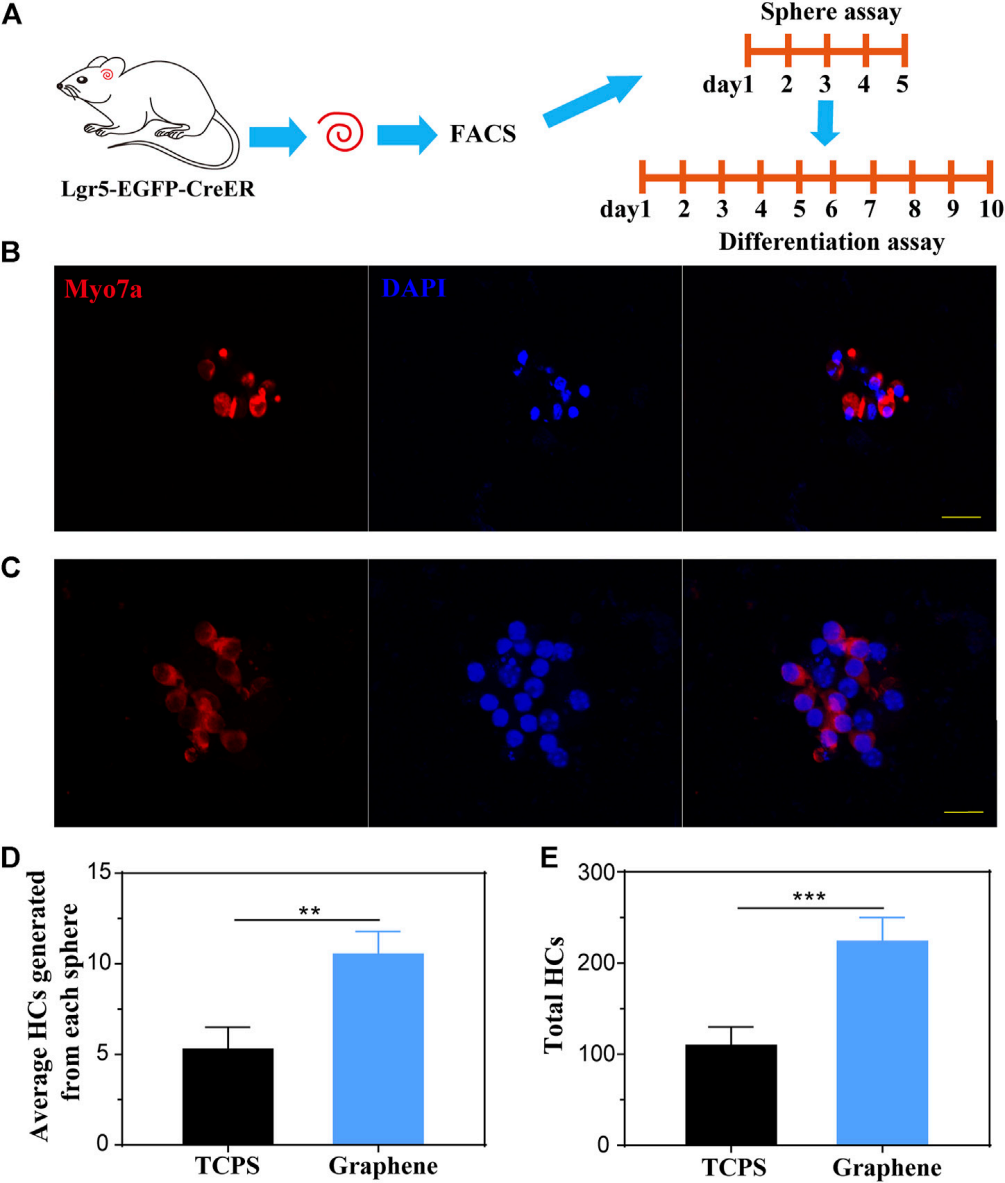
Lgr5+ spheres cultured on graphene substrates generate more HCs compared to those on TCPS. **(A)** Lgr5+ progenitors were cultured on different substrates for 5 days of sphere assay and 10 days of differentiation assay. **(B,C)** An Lgr5+ sphere cultured on TCPS **(B)** and graphene substrates **(C)** stained with the Myosin7a (red) and DAPI (blue). **(D)** Quantification of the average number of HCs differentiated from each sphere. **(E)** Quantification of the total number of HCs differentiated from Lgr5+ progenitors per well. The scale bars are 20 μm in **(B,C)**.

To investigate the HC regeneration capability of the formed spheres on different substrates, we performed differentiation assay for 10 days after 5 days of sphere-forming assay, as shown in Figure 4A. After differentiation, the spheres were stained with HC marker Myosin7a (Figures 4B,C). Subsequently, we counted the number of Myosin7a+ cells generated from each sphere and total Myosin7a+ cells per well. It was suggested that each sphere cultured on graphene generated more Myosin7a+ HCs than sphere cultured on TCPS (Figure 4D), and the total spheres on graphene also generated more Myosin7a+ HCs than those cultured on TCPS (Figure 4E).

**FIGURE 5:**
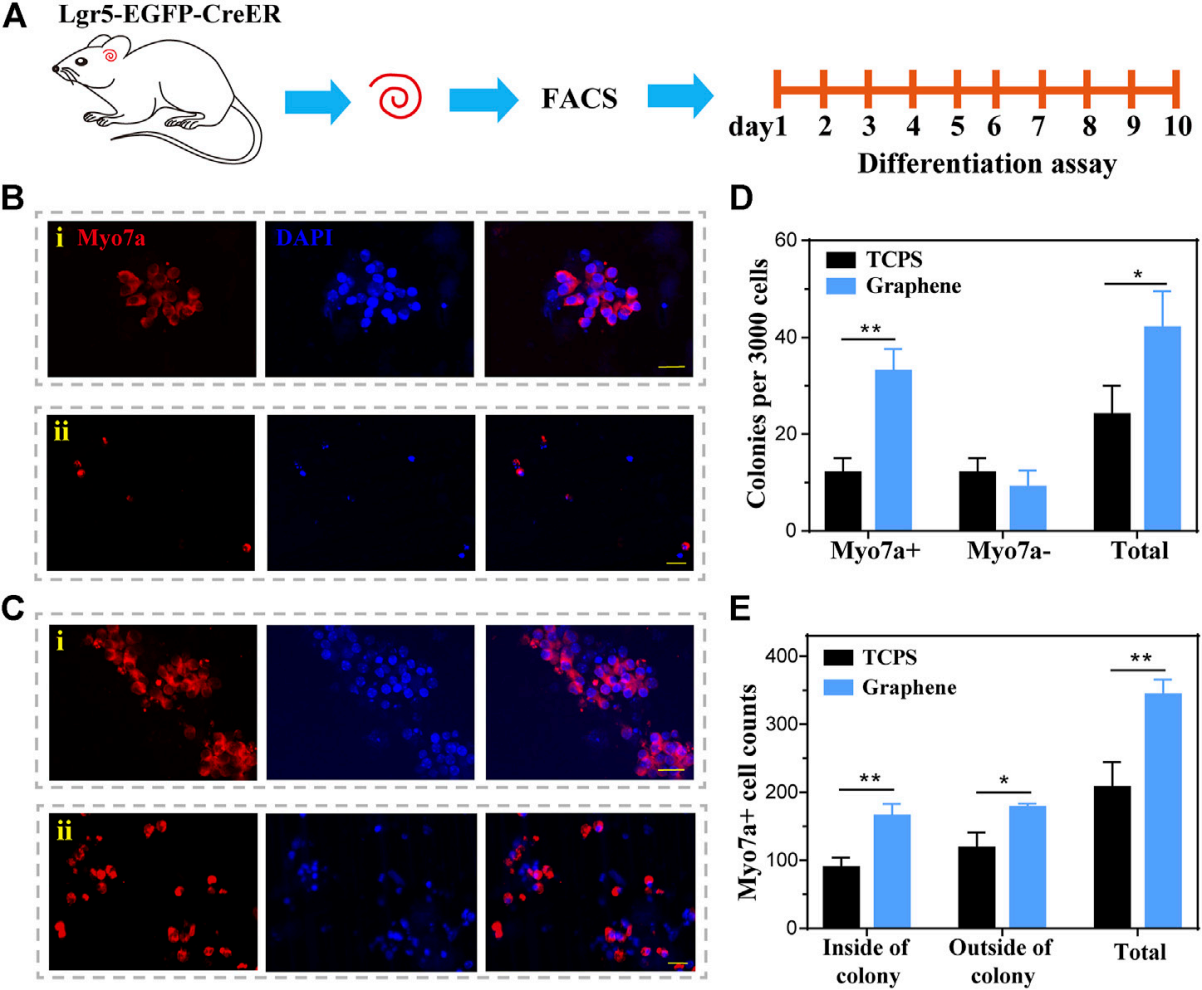
The differentiation of Lgr5+ progenitors. **(A)** The Lgr5+ progenitors were cultured on graphene or TCPS for 10 days of differentiation. **(B)** Immunofluorescence images of Lgr5+ progenitors cultured on TCPS after 10 days of differentiation, both inside (i) and outside (ii) the colony. **(C)** Immunofluorescence images of Lgr5+ progenitors cultured on graphene substrates after 10 days of differentiation, both inside (i) and outside (ii) the colony. **(D)** The number of colonies per 3,000 cells. **(E)** Quantification of Myosin7a+ cells. The scale bars are 20 μm in **(B,C)**.

To further explore the HC regeneration capability, we cultured the sorted Lgr5+ cells on laminin-coated graphene or TCPS substrates for 10 days of differentiation (Figure 5A). The cells were next stained with Myosin7a after 10 days of differentiation (Figures 5B,C). The results demonstrated that the cells cultured on graphene substrates formed significantly more Myosin7a+ colonies and total colonies than those cultured on TCPS (Figure 5D). It is worth noting that the Myosin7a+ cells inside the colony are mitotically regenerated HCs, and those outside the colony are directly differentiated HCs. The Myosin7a+ cells both inside and outside the colony were both counted. and the results suggested that the Lgr5+ progenitors cultured on graphene substrates differentiated more Myosin7a+ HCs both inside and outside the colony than those cultured on TCPS (Figure 5E). Overall, our findings suggest that graphene could promote the differentiation of Lgr5+ progenitors into HCs.

## Discussion

HCs in the mammalian cochlea mainly function for transduction of mechanical stimuli into electrical signals, thus play an important role in sound recognition. Irreversible damage or loss of HCs due to aging, ototoxic drugs, trauma, inflammation and other stress can lead to permanent hearing loss. In recent years, great progress has been made in HC regeneration, many studies have successfully induced embryonic stem cells and pluripotent stem cells to differentiate into hair cell-like cells *in vitro* (Roccio et al., 2018). Recently, several studies have reported that Lgr5+ SCs in the inner ear maintain the ability to generate HCs and SCs, and thereby considered inner ear progenitor cells. Lgr5 is a target gene of Wnt and is considered a stem cell marker in a variety of adult tissues (Barker et al., 2007; Jaks et al., 2008).

Recently, Smith-Cortinez et al. reported the long-term presence of Lgr5+ SCs in the adult mouse cochlea (Smith-Cortinez et al., 2021). These inner ear progenitor cells are able to survive in the cochlea even after severe ototoxic injury. Therefore, they have great potential in the treatment of sensorineural hearing loss caused by HC damage or loss. However, the efficiency of proliferation and regenerating HCs from Lgr5+ progenitors are low. Many studies have focused on the regulation of the proliferation and differentiation capacity of inner ear progenitor cells by different methods. With the development of tissue engineering technology, several novel therapeutic systems combining biomaterials with stem cells have been widely studied (Tong et al., 2015; Kim et al., 2018). In this work, we introduced graphene as a cell culture substrate to investigate its regulation on the behaviors of inner ear Lgr5+ progenitors, including proliferation and differentiation.

Graphene substrates have been shown to have great cytocompatibility and electrical conductivity, and have been extensively studied in the biomedical field. Various studies have suggested that graphene substrates could support cell culture and regulate the proliferation and differentiation of different types of stem cells (Park et al., 2011; Guo et al., 2016; Kenry et al., 2018). The regulation of stem cell differentiation by graphene opens a new horizon for its applications in regenerative medicine. However, its potential in the field of auditory field is remain exploring. Therefore, in this work, we investigated the *in vitro* regulation of graphene on inner ear progenitors, especially their proliferation and differentiation.

The sphere-forming assay suggested that Lgr5+ progenitors cultured on graphene substrates generated more spheres than those cultured on TCPS *in vitro*. However, there was no statistical difference in the diameter of the formed spheres. The results indicate that graphene can promote the sphere-forming ability of Lgr5+ progenitors. The results of differentiation assay suggested that Lgr5+ progenitors could differentiate into Myosin7a+ cells, and the number of Myosin7a+ cells on graphene was larger than TCPS, indicating that our prepared graphene substrates could promote the generation of HCs from Lgr5+ progenitors. Recent studies have found that the regeneration of HCs from inner ear progenitors were regulated by several genes, including Pou4f3, Atoh1, Cdh23, Jag2, Skp2 (Waqas et al., 2016). We speculate that graphene substrates may promote the differentiation of Lgr5+ progenitors into HCs by regulating the expression of related genes. We will carry out in-depth research on the underlying mechanism in the future. What’s important, we will develop more graphene-based 3D scaffolds with superior properties and combine them with stem cell transplantation for the regeneration of inner ear HCs.

In summary, we prepared graphene substrates by depositing graphene onto coverslips. We found that the graphene substrates promoted the sphere-forming and HC regeneration capabilities of cochlea Lgr5+ progenitors. The results suggest that graphene is an efficient interface that can promote the differentiation of Lgr5+ progenitor cells into HCs, which is essential for its future application in combination with Lgr5+ cells to regenerate HCs in the inner ear. In future work, we will continue to explore better graphene-based scaffolds to advance the application of graphene in stem cell therapy. In addition, we plan to further explore the specific mechanism by which graphene substrates regulate cell proliferation and differentiation. Understanding the specific mechanism of the interaction between graphene and inner ear Lgr5+ progenitors may provide more information for promoting HC regeneration through tissue engineering approaches.

## Data Availability

The raw data supporting the conclusion of this article will be made available by the authors, without undue reservation.
