# Supplementary figures and images for: Graphene Substrates Promote the Differentiation of Inner Ear Lgr5+ Progenitor Cells Into Hair Cells
Source: Front Bioeng Biotechnol. 2022 Jun 22;10:927248. doi: 10.3389/fbioe.2022.927248 (PMC9256972; doi:10.3389/fbioe.2022.927248)

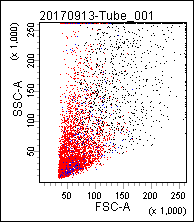

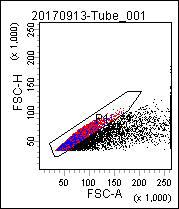

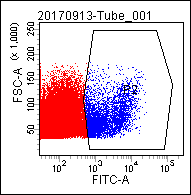

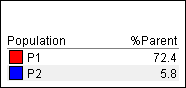

Supplement: Supplementary file 1 [file DataSheet1.ZIP › raw data/figure2/flow sorting/flow.docx]

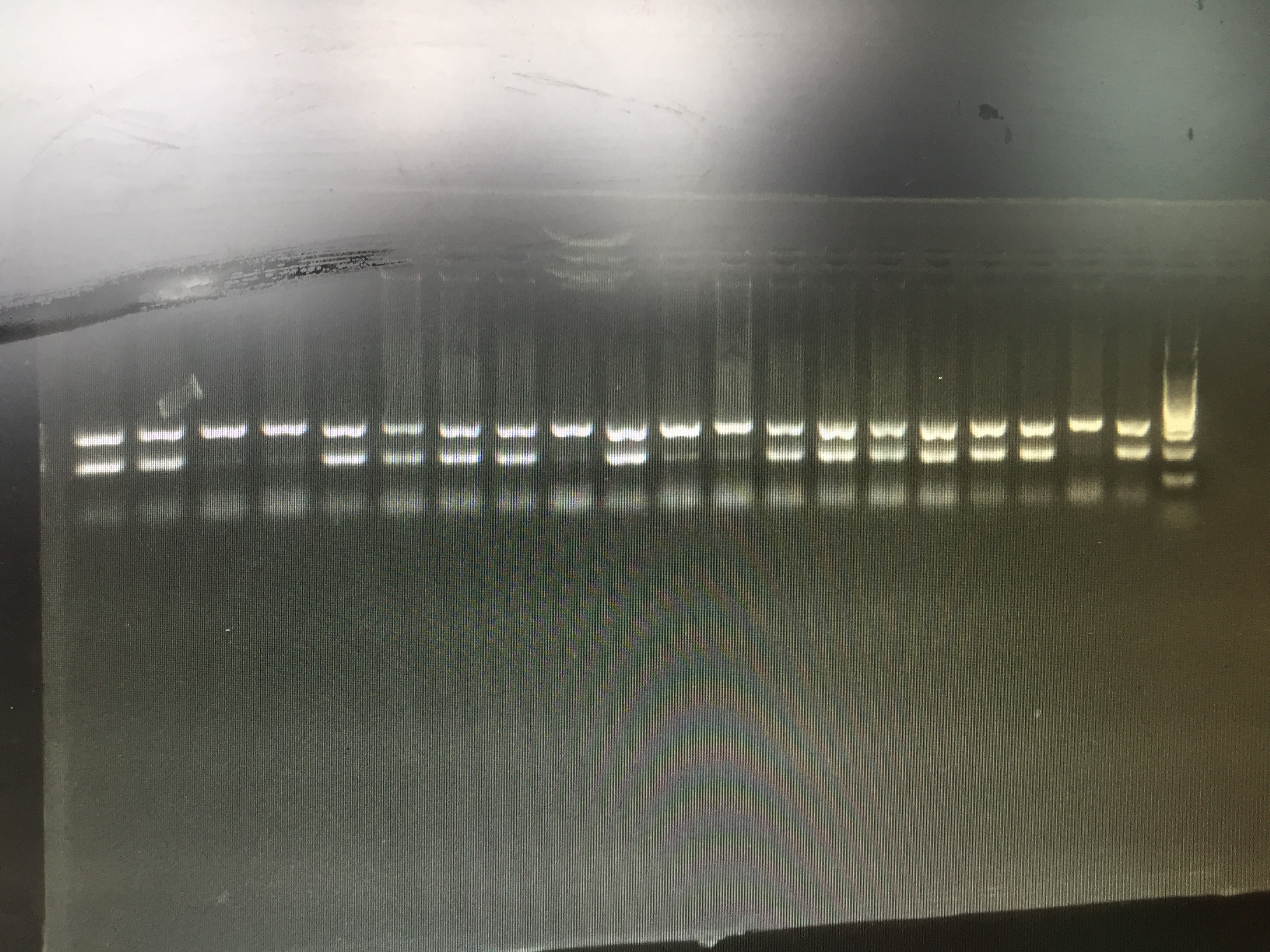

Supplement: Supplementary file 1 [file DataSheet1.ZIP › raw data/genotype gel.JPG]
